# Precise Detection of Surgical Margin in Head and Neck Cancer Using Dual Near-Infrared Imaging of the Tumor and Tumor Microenvironment

**DOI:** 10.34133/bmr.0235

**Published:** 2025-11-21

**Authors:** Kyu Young Choi, Hae Sang Park, Swarali Paranjape, Lauren Dang, Paul Jang, Jinhui Ser, Atsushi Yamashita, Kai Bao, Chan Hum Park, Satoshi Kashiwagi, Hak Soo Choi

**Affiliations:** ^1^ Gordon Center for Medical Imaging, Department of Radiology, Massachusetts General Hospital and Harvard Medical School, Boston, MA 02114, USA.; ^2^ Department of Otorhinolaryngology-Head and Neck Surgery, Kangnam Sacred Heart Hospital and Hallym University College of Medicine, Seoul 07441, South Korea.; ^3^ Department of Otorhinolaryngology-Head and Neck Surgery, Chuncheon Sacred Heart Hospital and Hallym University College of Medicine, Chuncheon 24252, South Korea.; ^4^ School of Materials Science, Japan Advanced Institute of Science and Technology, Ishikawa 923-1292, Japan.

## Abstract

The precise determination of resection margins during head and neck cancer surgery remains an unmet clinical challenge, where balancing complete tumor removal with preservation of healthy tissue is critical. To address this, we developed a dual near-infrared (NIR) fluorescence imaging strategy targeting both tumor cells and the tumor microenvironment (TME) in head and neck squamous cell carcinoma (HNSCC). Armed with 2 small-molecule fluorophores, OCTL14 for tumor-specific imaging and cRGD-ZW800-PEG for TME visualization, we performed real-time intraoperative NIR imaging in a FaDu tongue cancer xenograft model. Fluorophores were administered intravenously, and their targeting efficiency was quantified via time-dependent tumor-to-background ratios (TBRs), with surgical margins validated by histopathology. Our results demonstrated robust detection of cancerous tissue (TBR > 2.0) and surrounding TME (TBR > 1.5) within 4 h post-injection. Histopathology confirmed OCTL14 uptake in tumor cells, while cRGD-ZW800-PEG localized to peritumoral regions and vasculature. This dual-imaging approach offers a promising tool for fluorescence-guided surgery, enabling precise margin delineation to reduce locoregional recurrence and perioperative complications, thereby improving patient outcomes and quality of life.

## Introduction

Head and neck squamous cell carcinomas (HNSCCs) refer to cancers derived from the mucosal epithelium in the oral cavity, oropharynx, hypopharynx, and larynx [1,2]. Squamous cell carcinoma (SCC) comprises more than 90% of cancer cell types in the head and neck region [3]. The standard treatment of curative therapy for locally or locoregionally confined HNSCC is surgery, radiation, chemotherapy, or combination therapy [2,4]. Since treatment differs according to the stage of disease, primary site, and surgical accessibility, the role of clinical examinations and radiologic assessments in accurate treatment planning of this disease cannot be overstated. These assessments play an important role in guiding the treatment of HNSCC patients and thus impacting their survival [5]. As a result, most patients nowadays take extensive clinical and radiologic workup, including computed tomography (CT), magnetic resonance imaging (MRI), and fluorodeoxyglucose positron emission tomography/computed tomography (FDG-PET/CT).

While surgical removal of cancerous tissue presents the best overall survival rate for HNSCC, with the tumor-free margin being an important prognostic factor, resection margins of head and neck cancers such as oral SCCs are often inadequate (positive margins rate reported up to 15%) due to the complicated anatomy of the head and neck region [3,6,7]. Preoperative imaging studies, such as CT, MRI, and FDG-PET/CT, are not consistently available in the operating room, highlighting the urgent need for improved techniques. In vivo imaging can offer valuable insights into factors such as primary location, tumor margins, and the unforeseen extent of the disease. However, such real-time, precise imaging technology to determine resection margins while sparing healthy tissue during surgery for head and neck cancer is not currently available. Currently, intraoperative ultrasonography (US) and MRI can be used for real-time in vivo guidance in oral SCC. However, the sensitivity of these techniques is insufficient for identifying margins of less than 5 mm [8] and lacks information about the bony margins. As a result, the standard surgical procedure still relies on visual inspection and physical palpation by surgeons and frozen-section analysis, which is the only broadly accepted form of intraoperative margin assessment, but cannot give timely information within the surgical field and is also time and effort consuming. Thus, a novel technology that can determine the tumor-free margin intraoperatively is a major unmet clinical need.

Optical imaging, particularly fluorescence-guided surgery (FGS), is a promising technology for enhancing cancer visualization during surgery. FGS has emerged as a technique that not only provides real-time surgical guidance for surgeons to resect tumors but also minimizes normal tissue damage, thereby conferring a substantial benefit to patients [9,10]. FGS has been used to identify tumor tissues and surgical margins and proved to improve tumor resection rate and prognosis [11–13]. Various tumor-targeting molecules, such as antibodies, nanoparticles, proteins, peptides, and small molecules, have been developed for FGS. Among these molecules, anti-epidermal growth factor receptor (EGFR) antibodies labeled with Cy5.5 or IRDye800CW have been frequently employed to visualize HNSCC [14–17]. Current studies suggest that using these anti-EGFR-based fluorophores can be considered well tolerated in FGS for HNSCC regarding margin assessment, detection of metastatic lymph nodes, or second primary lesions [15–17]. However, antibody-targeting molecules are usually too large to transfer into the tumor and exhibit slow clearance, resulting in a low target-to-background ratio (TBR) and prolonged waiting times after administration (2 to 4 d), necessitating another hospital visit [18].

Small molecules, in contrast, which measure 10- to 1,000-fold smaller than peptides and proteins, quickly reach their target in vivo, and unbound molecules are rapidly cleared from the system, achieving the high signal-to-background ratio (SBR) promptly [18]. Due to the number of advantages, a small molecule has been optimized for intraoperative imaging. A targeted near-infrared (NIR) fluorophore conjugated to a ligand or with endogenous affinity to target tissue/cells (“structure-inherent targeting”) allows for specific tissue targeting compared with conventional nontargeted agents, such as indocyanine green (ICG) [19,20]. Such targeted molecules not only aid in identifying tumor margins during surgery but also enable identifying metastatic lymph nodes with the tumor cells, showcasing their potential in surgical applications. A small-molecule sensing chemical properties of the tumor microenvironment (TME), including mildly acidic conditions, has been developed for rapid in situ tumor imaging [21]. The rapid-acting properties of small molecules could be suitable for real-time determination of surgical margins.

However, surgical margin detection of head and neck cancer with a single agent has been challenging. For example, recent Phase II clinical trial results show notable discrepancies in sensitivity, specificity, and positive or negative predictive value of cetuximab-800CW depending on arbitrary cutoff values with SBR 1.5 or 2.0 [22]. Since the safe surgical margin in HNSCC ranges from 1 to 2 mm to 10 to 15 mm depending on the primary site [23,24], the precise determination of the margin is critical for complete resection and sparing normal tissue considering the limited area between major organs and proximity to vital structures such as vessels and nerves in the head and neck area. This result highlights the difficulty of determining surgical margins with the current FGS modality, which relies on a single-channel fluorescence [22].

**Fig. 1.**
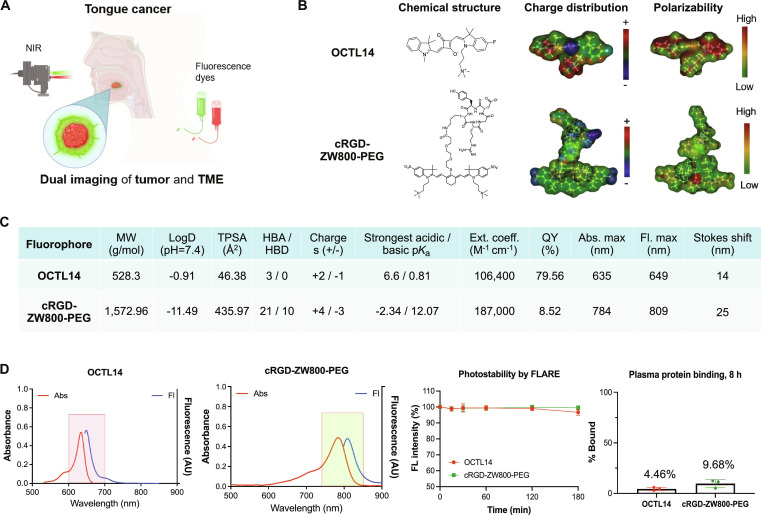
Chemical structure, physicochemical, and optical properties of OCTL14 and cRGD-ZW800-PEG for dual fluorescence imaging of HNSCC. (A) Schematic diagram of an oral SCC dual fluorescence imaging using OCTL14 and cRGD-ZW800-PEG. (B) Chemical structures of OCTL14 and cRGD-ZW800-PEG. (C) Physicochemical properties of OCTL14 and cRGD-ZW800-PEG. (D) Optical properties of OCTL14 and cRGD-ZW800-PEG in phosphate-buffered saline (pH 7.4) with 5% BSA, photostability patterns under 630- or 760-nm NIR for 4 h of incubating each fluorophore at 25 μM in 10% FBS, and plasma protein binding test results after 8 h of incubation. MW, molecular weight; TPSA, topological polar surface area; HBA, hydrogen bond acceptors; HBD, hydrogen bond donors; Ext. coeff., extinction coefficient; QY, quantum yield; Abs, absorbance; Fl, fluorescence. The schematic was drawn using BioRender.com.

To overcome this challenge, in this study, we established cutting-edge dual-channel NIR fluorescence imaging technology using tumor- and TME-targeted small-molecule NIR fluorophores for accurate assessment of surgical margin in HNSCC (Fig. 1A). A squaraine fluorophore, OCTL14, and an integrin αvβ3-targeted cRGD-ZW800-PEG were intravenously administered simultaneously to target tumor cells and TME, respectively. Within 4 h, the real-time NIR fluorescence imaging system, equipped with 2 independent NIR channels (700 and 800 nm) and color imaging, provided simultaneous fluorescence imaging to locate the tumor and TME in an orthotopic tongue cancer and an ectopic subcutaneous HNSCC xenograft model in mice. The fluorescence imaging of both tumor and TME provided a clear delineation of soft tissue surgical margin from adjacent normal tissue, enabling real-time imaging and surgical guidance of HNSCC on both models with a small tumor (<5 mm). In vitro cell survival studies and in vivo biodistribution studies demonstrated the safety of these fluorophores used in the dual fluorescence imaging of HNSCC. This dual-channel fluorescence imaging technology may overcome the historical challenge in FGS upon determination of surgical margins.

## Materials and Methods

### Synthesis of OCTL14 and cRGD-ZW800-PEG

All the reagents were obtained commercially from Fisher Scientific (Pittsburgh, PA, USA) or Sigma-Aldrich (St. Louis, MO, USA). Squaraine fluorophore OCTL14 was synthesized as previously described [25]. Briefly, alkylation of indole substrate with (3-bromopropyl) trimethylammonium bromide (BrTMAB) under reflux in acetonitrile (ACN) afforded the 3*H*-indolium salt derivative. Similarly, alkylation of the corresponding indole substrates under reflux in ACN afforded the 1,2,3,3-tetramethyl-3*H*-indolium iodide salts. Then, a squaric acid with isopropoxy protecting groups was treated to react under basic conditions, yielding the desired semi-squaraines. The semi-squaraines were reacted with the corresponding salt in a mixture of butanol and benzene containing quinoline as a base and heated under reflux using a Dean–Stark apparatus to furnish the asymmetric fluorinated squaraines.

The purity of all compounds was assessed using both ^1^H- and ^13^C-nuclear magnetic resonance (NMR) spectroscopy, as well as liquid chromatography–mass spectrometry (LC-MS) with a Waters Alliance e2695 separation module, a 2998 PDA detector (212 to 800 nm), and an Acquity QDA detector (*m*/*z* range: 50 to 1,239). LC-MS analysis was performed using an XBridge C18 reverse-phase high-performance liquid chromatography (HPLC) column (4.6 × 150 mm, 5 μm, Waters). Final compounds were purified via preparative HPLC (Waters 1525 Binary HPLC pump with a 2489 UV/Vis detector and an XBridge Prep C18 column, 19 × 150 mm, 5 μm). The collected fractions were concentrated by rotary evaporation and dried under vacuum overnight. For solvent purification, open column chromatography was used with 60 to 200 μ, 60 A, classic column silica gel (Dynamic Adsorbents, Norcross, GA). High-resolution accurate mass spectra (HRMS) were acquired using a Waters Micromass LCT TOF ES+ Premier Mass Spectrometer. The purity of the synthesized OCTL14 was revealed to be >95% (Fig. S1). cRGD-ZW800-PEG was also synthesized as previously described [26]. Briefly, the *N*-hydroxysuccinimide (NHS) ester form of ZW800-PEG was conjugated with cyclic RGDyK (cRGD, Arg-Gly-Asp-D-Tyr-Lys) in the presence of triethylamine in dimethyl sulfoxide. After facile and efficient solvent purification, cRGD-ZW800-PEG was obtained without the need for column chromatography (purity > 90%; Fig. S2).

### Physiochemical and optical properties

To evaluate the physicochemical properties of OCTL14 and cRGD-ZW800-PEG, molecular weight, partition coefficient (log*D* at pH 7.4), topological polar surface area (TPSA), hydrogen bond acceptors/donors (HBA/HBD), surface molecular charge, and the acid/basic dissociation constant (p*K*_a_) were calculated using MarvinSketch and JChem software (ChemAxon, Budapest, Hungary). To calculate the percentage of the bound (%Bound) for each compound, a rapid equilibrium dialysis device (Thermo Fisher, Waltham, MA, USA) was used to perform the serum protein binding assay. After 8 h of sample incubation (concentration of 5 μM) with 10% fetal bovine serum (FBS), Cytation 5 (BioTek, Winooski, VT, USA) was used to measure the absorbance of the fluorophores at the sample chamber and the buffer chamber to calculate the percentage of protein-bound samples. Fluorescence quantum yield (QY) was measured using oxazine 725 in ethylene glycol (QY 19%) for OCTL14 as a calibration standard under the condition of matched absorbance at 635 nm, and ICG in FBS (QY 10.1%) as a reference for cRGD-ZW800-PEG at 765 nm. The absorbance and fluorescence emission spectra were measured in 5% bovine serum albumin (BSA) using a USB-ISS-UV/VIS spectrophotometer (Ocean Optics, Dunedin, FL, USA). The molar extinction coefficient of OCTL14 and cRGD-ZW800-PEG was determined based on the Beer–Lambert law at 635 and 784 nm, respectively.

### Cell binding assay

To evaluate the cellular uptake of OCTL14 and cRGD-ZW800-PEG in HNSCC cells, a cell binding assay was done using FaDu cells (HTB-43), purchased from the American Type Culture Collection (Manassas, VA, USA). The cells were first cultured in complete Dulbecco’s modified Eagle’s medium (cDMEM) supplemented with 4.5 g/l glucose, l-glutamine and sodium pyruvate, 10% FBS, and 1% penicillin/streptomycin (Lonza, DE17-602E) at 37 °C in a 5% CO_2_ humidified chamber. Then, cells were seeded in a 24-well plate at a density of 20,000 cells/well and incubated for 24 h. After discarding old medium and washing twice with Hanks’ balanced salt solution (HBSS), the cells were incubated with OCTL14 for 20 min and with cRGD-ZW800-PEG for 30 min, respectively. The wells were washed 3 times with HBSS before fluorescence images were captured using Cy5 and Cy7 filters on Cytation 5 for OCTL14 and cRGD-ZW800-PEG, respectively.

### Cell viability assay

To evaluate the toxicity of OCTL14 and cRGD-ZW800-PEG on FaDu cells, cell viability was measured by using the Cell Counting Kit-8 (CCK-8, Dojindo Molecular Technologies Inc., Kumamoto, Japan). FaDu cells were seeded at a density of 400 cells/well in a 96-well plate, and then they were then treated with either 2, 5, 10, and 20 μM OCTL14 or 1.6, 3.1, 6.3, 12.5, 25, 50, and 100 μM cRGD-ZW800-PEG in growth medium for 24 h at 37 °C. Then, the dye and old medium were removed from all wells, and new medium were added and incubated for 24 h before 10 μl of CCK-8 solution was added to each well. After 3 h of incubation for OCTL14 and 4 h of incubation for cRGD-ZW800-PEG, absorbance was measured at 450 nm using a microplate reader. The cell survival rate for each dye was calculated using the following equation: Survival rate (%) = (Asample − Ab)/(Ac − Ab) × 100 (Asample, absorbance of sample; Ab, absorbance of blank well; Ac, absorbance of negative control). Experiments were repeated at least 3 times.

### Cellular uptake assay

To reveal the uptake mechanism of OCTL14 on FaDu cells, a cellular uptake inhibition assay was performed using bromsulphthalein (BSP) (Sigma-Aldrich, St. Louis, MO, USA) and corticosteroid (Sigma-Aldrich). First, the 15,000 cells were plated per well on a 24-well plate. After incubation for 48 h at 37 °C, cells were washed twice with 0.5 ml of HBSS. Then, the cells were pretreated with 250 μM BSP for 5 min or 25 μM corticosteroid for 5 min, before they were incubated with 2 μM OCTL14 for 15 min at 37 °C. To rule out the possible diffusion of OCTL14 across the plasma membrane, FaDu cells were incubated with 2 μM OCTL14 at 4 °C for 15 min. Serum-free medium was added to the control group without any inhibitors. After washing, images were acquired by Cytation 5 using a Cy5 filter. ImageJ software version 1.52p was used to measure the fluorescent intensity of FaDu cells at each condition.

### Biodistribution and clearance

The biodistribution of OCTL14 and cRGD-ZW800-PEG was evaluated to define the pharmacokinetics of these fluorophores in mice. Strain nude mice (8 to 12 weeks, female) were purchased from Charles River Laboratories (Wilmington, MA, USA) and were housed in an Association for Assessment and Accreditation of Laboratory Animal Care (AAALAC)-certified facility at Massachusetts General Hospital under the Institutional Animal Care and Use Committee (IACUC) approval (#2016N000136). To minimize autofluorescence, mice were fed with chlorophyll-free mouse chow (VWR International, Radnor, PA, USA) 3 d prior to the imaging study. OCTL14 and/or cRGD-ZW800-PEG (100 nmol) was diluted in 5% BSA to yield a total volume of 100 μl and injected intravenously via retroorbital injection under isoflurane anesthesia. After a series of intraoperative fluorescence imaging, animals were sacrificed to excise and image the major organs, including the heart, lungs, liver, pancreas, spleen, kidneys, duodenum, intestine, abdominal muscle, and tongue ex vivo at 4 h post-injection.

NIR fluorescence images were obtained by using the K-FLARE imaging system [27]. The dual-NIR channel imaging system provides color images by white light (400 to 650 nm) and 2 independent fluorescence images at 700- and 800-nm channels. A 630-nm excitation for OCTL14 with a fluence rate of 2 mW/cm^2^ and a 760-nm excitation for cRGD-ZW800-PEG with a fluence rate of 4 mW/cm^2^ were used for 700- and 800-nm channels, respectively. For NIR merged images, 700- and 800-nm fluorescence images were pseudo-colored in red and green, respectively. The fluorescence intensity of each major organ and tongue was quantified using ImageJ. SBR was calculated using the following equation: SBR = fluorescence intensity of a region of interest/fluorescence intensity of muscle.

### Intraoperative fluorescence imaging of orthotopic and ectopic tongue cancer

To evaluate the targetability of OCTL14 and cRGD-ZW800-PEG on the orthotopic xenograft model of tongue cancer, 0.5 × 10^6^ of FaDu cells in a 30-μl mixture of saline and Matrigel (1:2 ratio) were injected subepithelially into the tip of the tongue using an insulin syringe. When the size of the tongue tumor reached 2 mm, dual fluorescence imaging was performed by injecting cRGD-ZW800-PEG and OCTL14 intravenously 4 h prior to imaging. For the ectopic xenograft model of HNSCC, 0.5 × 10^6^ of FaDu cells in a 50-μl mixture of saline and Matrigel (1:2 ratio) were injected subcutaneously on the lower back of the mouse. When the size of the ectopic tumor reached 3 mm, in vivo evaluation of the targetability of the 2 NIR fluorophores was performed. All the animal surgeries were performed under anesthesia with isoflurane.

### Tumor targeting and quantitative analysis

The real-time fluorescence intensity of OCTL14 and cRGD-ZW800-PEG at the orthotopic and ectopic tumors was compared with the surrounding nontumor region (background signal) to evaluate the TBR. After intraoperative imaging, the fluorescence intensity of the tumor tissue was acquired using ImageJ software and divided by the fluorescence intensity of the surrounding tissue to calculate TBR for both OCTL14 and cRGD-ZW800-PEG. Quantitative time-course assessment of both orthotopic and ectopic tongue cancer was performed for both OCTL14 and cRGD-ZW800-PEG at 1, 4, 6, and 24 h post-intravenous injection. At least 3 head and neck tumor models were evaluated for the quantitative analysis of TBR.

### Histological analysis

Tumor and TME targetability of OCTL14 and cRGD-ZW800-PEG were confirmed by histology analysis on the orthotopic HNSCC xenograft model. The tumor-bearing tongue was excised from the mouse after in vivo imaging of the tumors at 4 h after intravenous injection of the 2 fluorophores, and embedded in Tissue-Tek optimal cutting temperature compound (Sakura Finetek, Torrance, CA, USA). Frozen sections were acquired and placed on slides with a cut thickness of 10 μm using a cryostat (Leica, Germany). Fluorescence images were taken for both OCTL14 and cRGD-ZW800-PEG using Cy5 filter and Cy7 filter on Cytation 5, respectively. After dual fluorescence imaging, the tissue sections were stained with hematoxylin and eosin (H&E), and bright-field images of the orthotopic tongue cancer were acquired using Cytation 5.

### Statistical analysis

The data were expressed as mean and standard error of the mean. Statistical analysis was conducted using one-way analysis of variance (ANOVA) followed by Tukey’s multiple comparisons tests by Prism version 8 software (GraphPad, San Diego, CA, USA). A *P* value of <0.05 was considered significant.

## Results

### Chemical and optical properties of OCTL14 and cRGD-ZW800-PEG

As shown in Fig. 1B, the structure of OCTL14 includes the central oxocyclobutenolate ring with a quaternary ammonium cation and a fluorine atom (Fig. S1). This feature significantly increases the molar absorptivity and the QY, making OCTL14 a highly efficient tool for biological applications [25]. The physicochemical properties of OCTL14 show descent hydrophilicity (log*D* at pH 7.4 = –0.9) (Fig. 1C). The NIR fluorescence of OCTL14 shows excitation and emission maxima at 635 and 649 nm, respectively, making it compatible with the 700-nm NIR channel of the K-fluorescence-assisted resection and exploration (K-FLARE) imaging system (Fig. 1D). The high molar absorptivity (Ext. Coeff. = 106,400 M^−1^ cm^−1^) and the high QY (QY = 79.56%) contribute to the ultrahigh brightness of OCTL14 in vivo. The photostability of OCTL14, assessed by irradiating the fluorophore under 630-nm NIR light, shows its robust endurance, maintaining over 90% of its initial absorbance for up to 3 h (Fig. 1D). The low plasma protein binding of OCTL14 (4.46%) supports rapid distribution to target tissues after intravenous administration for cancer imaging (Fig. 1D) [28].

The chemical structure of cRGD-ZW800-PEG reveals the cancer-targeting cRGD motif linked to ZW800-PEG via conventional NHS ester chemistry (Fig. 1B and Fig. S2). The physicochemical property of cRGD-ZW800-PEG showed high hydrophilicity (log*D* at pH 7.4 = –11.49) and high polarity (TPSA = 435.97), implying high water solubility and reduced cell permeability of cRGD-ZW800-PEG (Fig. 1C). cRGD-ZW800-PEG exhibited absorbance and fluorescence spectra under 760-nm NIR light, enabling simultaneous dual-channel imaging of OCTL14 and cRGD-ZW800-PEG without spectral overlap (Fig. 1D). The photostability of cRGD-ZW800-PEG under 760-nm NIR light remained consistently over 99% up to 3 h, ensuring the feasibility in intraoperative imaging (Fig. 1D). The protein binding study of cRGD-ZW800-PEG revealed low plasma protein binding (9.68%) (Fig. 1D), owing to the balanced zwitterionic charge and the flexible PEG linker. Similar to OCTL14, the low serum binding is considered to support rapid distribution and targeting in tumor tissue [25,28].

**Fig. 2.**
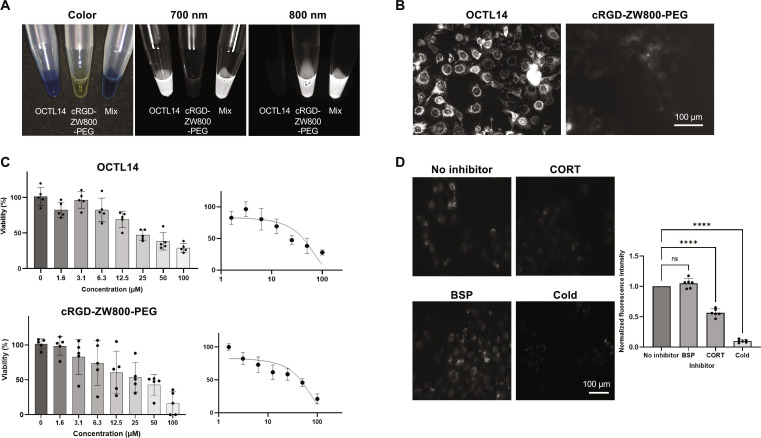
Cytotoxicity and cellular uptake of OCTL14 and cRGD-ZW800-PEG. (A) Color and fluorescence images of OCTL14 (10 nM) and cRGD-ZW800-PEG (10 nM) under NIR channel 700 nm and 800 nm. (B) Cellular binding assay of OCTL14 and cRGD-ZW800-PEG on FaDu cells. The images for OCTL14 and cRGD-ZW800-PEG were acquired under Cy5 and Cy7 channels in Cytation 5, respectively. (C) Cytotoxicity assay of OCTL14 and cRGD-ZW800-PEG in FaDu cells using CCK-8 assay, and dose-response curves of FaDu cells after logarithmic dosing of OCTL14 and cRGD-ZW800-PEG. IC_50_ values were as follows: OCTL14 = 42.12 μM, cRGD-ZW800-PEG = 45.82 μM. *n* = 5. (D) Cellular uptake inhibition assay of OCTL14 in FaDu cells. Cells were pre-blocked with BSP or corticosteroid (CORT) and then incubated with OCTL14. Cold inhibition was performed with incubation of 2 μM OCTL14 with FaDu cells at 4 °C for 30 min. ****P < 0.0001. *n* = 6 (B and D).

Following the result, we tested the feasibility of multi-channel NIR imaging using these fluorophores under the K-FLARE system. Figure 2A shows the color and fluorescence images of OCTL14, cRGD-ZW800-PEG, and both fluorophores in vitro, revealing the distinct fluorescence signal of OCTL14 under the NIR 700-nm channel and the fluorescence signal of cRGD-ZW800-PEG under NIR 800-nm channel. When equal concentrations (10 nM) for both OCTL14 and cRGD-ZW800-PEG were used to be excited by the corresponding NIR channels, the minimal overlapping fluorescence emission of these fluorophores was noted. These results suggest that the combination of OCTL14 and cRGD-ZW800-PEG is optimal for multi-channel, real-time NIR imaging.

### Cellular uptake and cytotoxicity

The uptake of OCTL14 and cRGD-ZW800-PEG in FaDu cells was demonstrated through a cell binding assay (Fig. 2B). The NIR images taken after incubation with the fluorophores revealed a significantly high target affinity of OCTL14 for FaDu cells, which is a favorable feature for cancer cell imaging. In contrast, cRGD-ZW800-PEG exhibited limited uptake in FaDu cancer cells with the given condition. Consistently, our previous study suggests that cRGD-ZW800-PEG mainly targets αvβ3 and other integrins in TME of HNSCC [29]. Next, the cytotoxicity of OCTL14 and cRGD-ZW800-PEG on FaDu cells was investigated using the CCK-8 assay. OCTL14 or cRGD-ZW800-PEG showed little toxicity up to a concentration of 20 or 25 μM, respectively (Fig. 2C). These results demonstrated the safety of these fluorophores in bioimaging and image-guided surgeries for HNSCC.

To determine the cellular uptake mechanisms of OCLT14 in HNSCC, the cellular uptake inhibition assay was performed in FaDu cells (Fig. 2D). We have previously shown that squaraine fluorophores were taken up via organic cation transporters (OCTs) [25]. Consistently, corticosteroid, known to inhibit OCTs [30], significantly reduced the uptake of OCTL14 compared to the control in FaDu cells. A small-molecule fluorophore can be taken up via organic anion-transporting polypeptides (OATPs) [31]. However, BSP, an established inhibitor of OATPs [31], had little impact on the cellular uptake of OCTL14 in FaDu cells. These findings indicate that OCTs play a dominant role in OCTL14 uptake in HNSCCs [30,32,33]. Negative uptake in the cold inhibition test further confirmed that the transport of OCTL14 into FaDu cells was receptor mediated, thereby ruling out the likelihood of passive diffusion of the substance across the plasma membrane.

### Biodistribution and tumor targetability

**Fig. 3.**
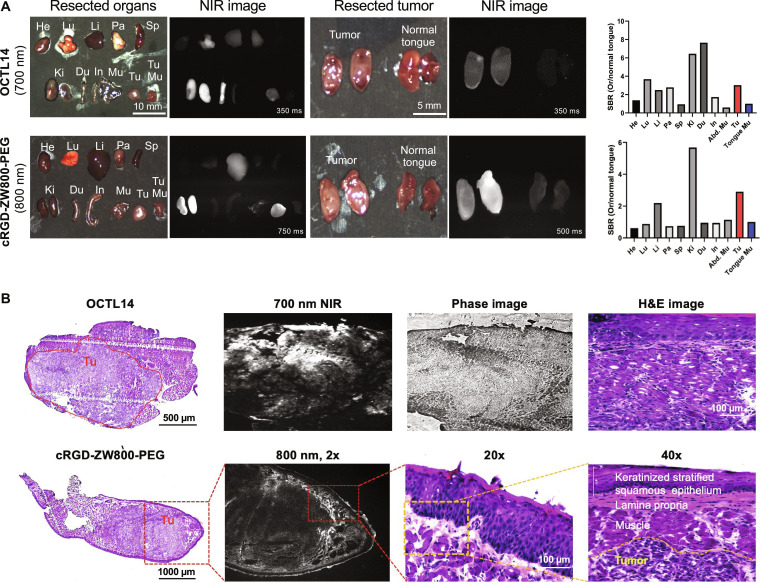
In vivo biodistribution and tumor targetability of OCTL14 and cRGD-ZW800-PEG. (A) Representative color and NIR images of the resected major organs and tongue tumor compared with normal tongue. NIR images for OCTL14 and cRGD-ZW800-PEG were taken 4 h after intravenous injection. The SBR was calculated using ImageJ and Prism 8 software. (B) Histopathologic and NIR images of cross-sectioned tongue tumor show tumor targeting of OCTL14, and longitudinal-sectioned tongue tumor shows tumor margin targeting of cRGD-ZW800-PEG. NIR, near-infrared; He, heart; Lu, lung; Li, liver; Pa, pancreas; Sp, spleen; Ki, kidney; Du, duodenum; In, intestine; Mu, muscle; Tu, tumor; SBR, signal-to-background ratio. SBR = fluorescence intensity of a region of interest/surrounding nontumor region (muscle tissue).

To determine the in vivo biodistribution of OCTL14 and cRGD-ZW800-PEG upon intravenous injection, 100 nmol of each fluorophore was administered to tumor-bearing mice, followed by intravital and ex vivo imaging using the K-FLARE system. Consistent with previous results of post-6 h in vivo biodistribution [25], OCTL14 showed high SBR in the kidney, gallbladder, and intestine (Fig. 3A), indicating hepatobiliary and renal clearance. On the other hand, cRGD-ZW800-PEG exhibited a high fluorescence signal in the kidney with low background tissue uptake, indicating rapid renal clearance and ensuring minimal nonspecific binding. Other organs showed minimal uptake except these organs. In addition to the results of ex vivo imaging, real-time in vivo imaging revealed that both OCTL14 and cRGD-ZW800-PEG accumulate in the kidneys and bladder, indicating efficient renal clearance of these fluorophores (Fig. S3). In addition, consistent with little toxicity observed in the FaDu cells during the in vitro tests, no obvious acute toxicity was noted during the in vivo tests upon administration of these fluorophores. These findings warranted the intravenous injection of the combination of OCTL14 and cRGD-ZW800-PEG for fluorescence imaging of tumors in vivo.

The HNSCC tongue cancer model was well established within 17 d after the injection of FaDu cells in nude mice. The orthotopic tongue tumor was successfully detected by both OCTL14 in the 700-nm and cRGD-ZW800-PEG in the 800-nm NIR channel (Fig. 3B). Histological analysis revealed a well-formed FaDu cell tumor confined to the intramuscular region, with well-preserved epithelium and lamina propria (Fig. 3B). Histopathologic analysis indicated that while OCTL14 effectively imaged the tumor cell itself, which is consistent with the in vitro uptake study (Fig. 2B), peritumoral tissue was the primary target for NIR fluorescence imaging by cRGD-ZW800-PEG, which is consistent with our previous study showing that cRGD-ZW800-PEG targets TME, including tumor vasculature in HNSCC.

### Dual intraoperative fluorescence imaging of HNSCC

**Fig. 4.**
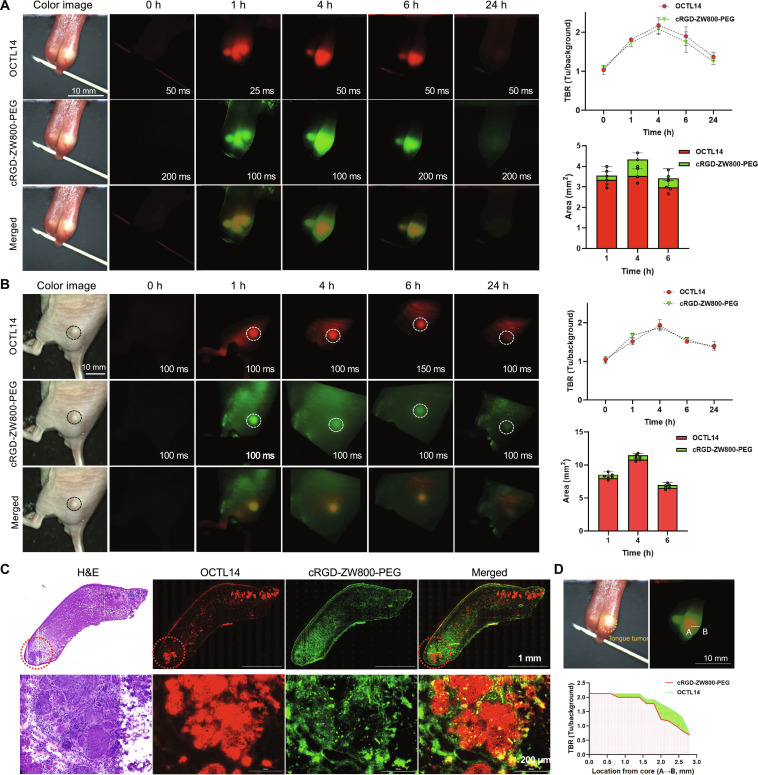
Intraoperative real-time dual imaging of an orthotopic tongue tumor model using OCTL14 and cRGD-ZW800-PEG. (A) Time-course measurements of fluorescence intensities at the tumor sites targeted by OCTL14 (red) and cRGD-ZW800-PEG (green), and quantitative fluorescence analysis of tumor-to-background (TBR) and targeted area for the orthotopic tumor model. (B) Time-course measurements of intensities at the tumor sites targeted by OCTL14 and cRGD-ZW800-PEG, as well as quantitative fluorescence analysis of TBR and targeted area for the ectopic tumor model. (C) Histopathological H&E and fluorescence images of FaDu cell tongue cancer using OCTL14 and cRGD-ZW800-PEG. SCC at the tip of the tongue in the orthotopic model was imaged by OCTL14 (red), and the surrounding TME was imaged by cRGD-ZW800-PEG (green), simultaneously under K-FLARE imaging system. TBR = fluorescence intensity of a region of interest/surrounding nontumor region (muscle tissue), calculated using ImageJ and Prism 8 software (*n* = 3). (D) Fluorescence intensity profiling of the tumor from core to periphery. The white dotted line from A to B indicates the tumor margin definition created by the TBR of OCTL14 and cRGD-ZW800-PEG.

To determine the effectiveness of the dual fluorescence imaging for HNSCC, the combination of 100 nmol of OCTL14 and cRGD-ZW800-PEG was administered to the orthotopic tongue and subcutaneous mouse models of HNSCC, followed by intravital and ex vivo imaging using the K-FLARE system. The real-time intraoperative color and NIR fluorescence images shown in Fig. 4 demonstrate the fluorescence signal of OCTL14 aligned with the tongue (Fig. 4A) and subcutaneous tumors (Fig. 4B). Histological analysis revealed that the OCLT14 signal was precisely colocalized with the tumor area, effectively avoiding adjacent tissues (Fig. 4C). In contrast, the signal from cRGD-ZW800-PEG was detected in a broader area, extending beyond the tumor (Fig. 4A and B). Histological analysis confirmed that cRGD-ZW800-PEG was located in TME and peritumor tissue of HNSCC (Fig. 4C). Positive fluorescence of cRGD-ZW800-PEG was also detected in small tumor nests embedded in normal tissue. Thus, this combination effectively targeted the tumor and TME independently in their respective NIR channels, showing minimal crosstalk between the 700- and 800-nm channels. The TBR was higher than 1.5 for each fluorophore, continued to increase until 4 h post-injection, peaking at that time, and remained higher than 1.5 up to 6 h post-injection in both the orthotopic and subcutaneous tumor models (Fig. 4A and B). The distribution of the signal, showing tumor targeting with OCTL14 and TME with cRGD-ZW800-PEG, remained consistent throughout the imaging window (Fig. 4A and B). This suggests that a single simultaneous intravenous injection of the combination of these fluorophores is capable of maintaining their efficacy in real-time imaging throughout the entire surgical procedure of HNSCC surgeries. A line distribution analysis of the fluorescence signal from the core to the periphery of the tumor revealed a clear cancer core, imaged with OCTL14, surrounded by the TME, imaged with the cRGD-ZW800-PEG signal (Fig. 4D), demonstrating the real-time mapping of a tumor and its TME within the imaging field and providing a clear and robust delineation of the tumor margin. These results together suggest that dual fluorescence imaging is a feasible approach to define surgical margins of HNSCC.

## Discussion

Tumor recurrence is a significant factor affecting mortality in HNSCC, with 10% to 30% of patients experiencing locoregional recurrence even after definitive treatment with tumor-free resection margins [34,35]. In a recent clinical phase II trial study, 14 tumor-positive margins were found in 66 oral SCCs after surgical resection with a 1-cm clinical margin, which led to postoperative chemotherapies and radiotherapies with increased morbidities [22]. Establishing clear surgical margins at the time of initial surgery can minimize the need for reoperation and adjuvant therapies; however, overlying aggressive resection of normal tissue may lead to substantial functional and cosmetic deficits, negatively impacting the patient’s quality of life. Therefore, precise margin assessment is essential to balance oncologic control with tissue preservation. To address this challenge, technologies for more precise determination of surgical margins have been actively pursued.

In current clinical settings, histological analyses of frozen biopsies from the surgical margins are typically conducted to identify potential tumor remnants. However, cancer cells can evade detection due to undersampling when only a small number of cancer cells are present in a large tissue volume, potentially leading to incomplete treatment of the cancer. Fluorescence-guided assessment of tumors enables more accurate surgical margins in the operative field compared to the surgeon’s naked eye, inducing more complete tumor removal and subsequently enhancing patient survival, which is the ultimate goal of this study. Although no targeted NIR fluorophores for image-guided surgery of HNSCC have yet been approved for clinical use, a number of agents for NIR fluorescence imaging have been introduced to head and neck cancer surgeries recently, including PARPi-FL, cRGD-ZW800-1, and panitumumab-IRDye800, and tested in clinical trials [36].

In the current study, we demonstrated the feasibility of a novel technology to precisely and robustly determine surgical margins with multichannel NIR fluorescence imaging using tumor- and TME-targeted fluorophores. A squaraine fluorophore, OCTL14, has been developed and proven efficient and safe in achieving rapid and durable imaging of ovarian cancer in our previous study [25]. OCTL14 was taken up by the target tissue via OCTs [25], which are also reported to be highly expressed in HNSCCs [32,33,37]. cRGD is well known as a targeting ligand for specific binding to the integrin receptor (αvβ3 subunit) overexpressed in various cancer cell types, including HNSCC [29]. While FaDu cells express little integrin αvβ3, we have demonstrated that cRGD-ZW800-PEG can detect αvβ3-positive intratumoral HNSCC due to the RGD’s affinity to the angiogenic tumor tissue [38]. These findings explain the safety and the positive targeting of cRGD-ZW800-PEG on TME (e.g., tumor vasculature) of FaDu cell tumor [29]. In this study, for the first time, we established an imaging method to achieve clear delineation and mapping of the localization of tumor cells and TME with high TBR after a single intravenous injection of the combination of tumor-targeted OCTL14 and TME-targeted cRGD-ZW800-PEG (Fig 4). There was no apparent overlap of each fluorescence signal, and operators could observe merged images in real-time multichannel imaging using the K-FLARE imaging system. The clear delineation created by this dual imaging technology can offer enhanced surgical margin definition and detection in HNSCC surgery, compared to a surgical margin created by a single fluorescence imaging with a possibly obscure TBR cutoff. The real-time mapping of a tumor and TME would help surgeons assess the surgical margin more efficiently and determine the resection area while ensuring that a positive or close margin is a critical prognostic factor. Thus, this technology is expected to improve the determination of surgical margins during intraoperative imaging and provide accurate surgical removal of both the tumor and its TME by FGS.

The complete removal of tumor buds may reduce cancer relapse and increase the survival rate of HNSCC patients in clinics. Enhanced margin definition by the dual-imaging strategy can provide practical surgical guidance, including robotic or artificial intelligence-assisted surgeries. However, the effect of this imaging strategy on enhancing HNSCC patients’ survival needs to be clarified through clinical trials, a crucial step in validating the findings of this study. If proven effective, while the frozen biopsy method is currently used for the intraoperative margin assessment, the paradigm in oncologic surgeries can be changed to the FGS imaging of both tumor and TME for safety margin assessment. Overall, this dual-channel imaging strategy holds promise to revolutionize HNSCC detection and surgery through rapid detection, feasibility of use, safety, and cost-effectiveness.

To conclude, the combination of tumor-targeted OCTL14 and TME-targeted cRGD-ZW800-PEG presents a novel dual-imaging strategy for intraoperative imaging of HNSCC for precise surgical margin detection and determination. This approach efficiently targets the tumor and its margins with TME, using 2 independent wavelengths of NIR fluorescence. The fluorescence imaging of the tumor and the tumor margin provides a clear and robust delineation of tumor margins and informed surgical guidance in tumor resection, compared to the obscure tumor margin by a single fluorescence imaging of the tumor only. This technology is featured with minimal nonspecific uptake and rapid clearance from the body upon intravenous injections for safety and effective imaging, holding the potential for clinical application in detecting cancerous areas in the surgical field of HNSCC patients. These fluorescence agents are amenable to current Good Manufacturing Practices (cGMP) using a streamlined production protocol and can be synthesized economically, thus being suitable for clinical use. This technology is expected to be more broadly applicable to other cancer surgeries and have a significant impact in reducing mortality in cancer patients.

## Ethic Approval

All animal procedures were performed in accordance with the Public Health Service Policy on Humane Care of Laboratory Animals and approved by the MGH IACUC (#2016N000136).

## Data Availability

The datasets and materials used and/or analyzed during the current study are available from the corresponding author upon reasonable request.
