# Supplementary material for: Precise Detection of Surgical Margin in Head and Neck Cancer Using Dual Near-Infrared Imaging of the Tumor and Tumor Microenvironment
Source: Biomater Res. 2025 Nov 21;29:0235. doi: 10.34133/bmr.0235 (PMC12636036; doi:10.34133/bmr.0235)
Supplement: Supplementary 1 — Figs. S1 to S3 [file bmr.0235.f1.docx]

**Supporting Information**

**Precise detection of surgical margin in head and neck cancer using dual near-infrared imaging of the tumor and tumor microenvironment**

Kyu Young Choi^1,2†^, Hae Sang Park^1,3†^, Swarali Paranjape^1^, Lauren Dang^1^, Paul Jang^1^, Jinhui Ser^1^, Atsushi Yamashita^1^, Kai Bao^1^, Chan Hum Park^3,*^, Satoshi Kashiwagi^1,*^, Hak Soo Choi^1,4,*^

^1^Gordon Center for Medical Imaging, Department of Radiology, Massachusetts General Hospital and Harvard Medical School, Boston, MA 02114, USA.

^2^Department of Otorhinolaryngology-Head and Neck Surgery, Kangnam Sacred Heart Hospital and Hallym University College of Medicine, Yeongdeungpo-gu, Seoul 07441, South Korea.

^3^Department of Otorhinolaryngology-Head and Neck Surgery, Chuncheon Sacred Heart Hospital and Hallym University College of Medicine, Chuncheon 24252, South Korea.

^4^School of Materials Science, Japan Advanced Institute of Science and Technology, 1-1 Asahidai, Tatsunokuchi, Ishikawa 923-1292, Japan

†These authors contributed equally to this work.

*Correspondence to: skashiwagi@mgh.harvard.edu (S.K.); hlpch@paran.com (C.H.P.); mghchoilab@gmail.com (H.S.C.).

**Table of Contents**

**Figure S1.** ^1^H- and ^13^C-NMR spectroscopy and HPLC-MS spectrometry of OCTL14.

**Figure S2.** ^1^H- and ^13^C-NMR spectroscopy and HPLC-MS spectrometry of cRGD-ZW800-PEG.

**Figure S3.** Real-time NIR fluorescence imaging of OCTL14 and cRGD-ZW800-PEG in major organs and tongue tumor.

**
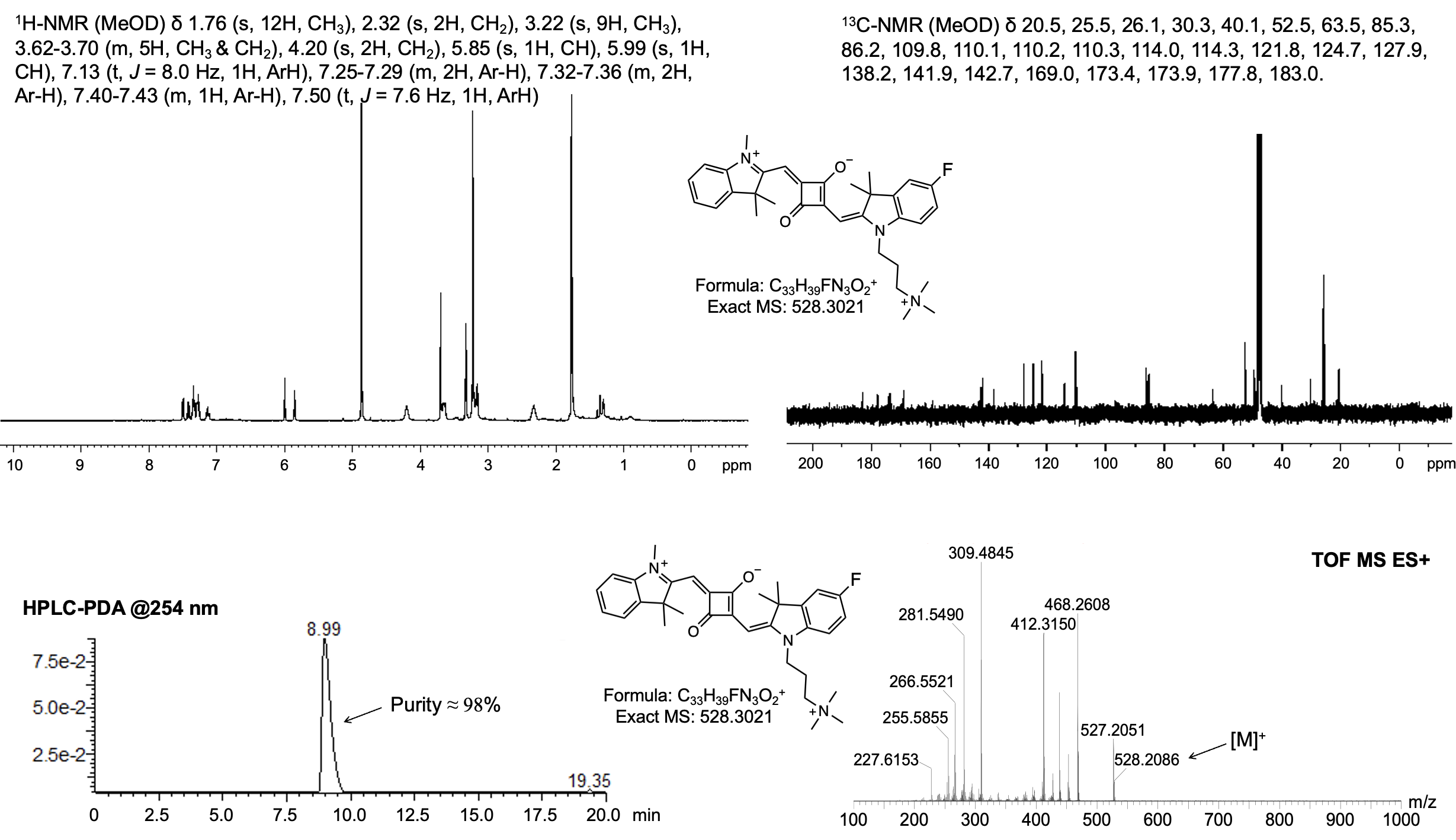
**

**Figure S1.** ^1^H- and ^13^C-NMR spectroscopy and HPLC-MS spectrometry of OCTL14.


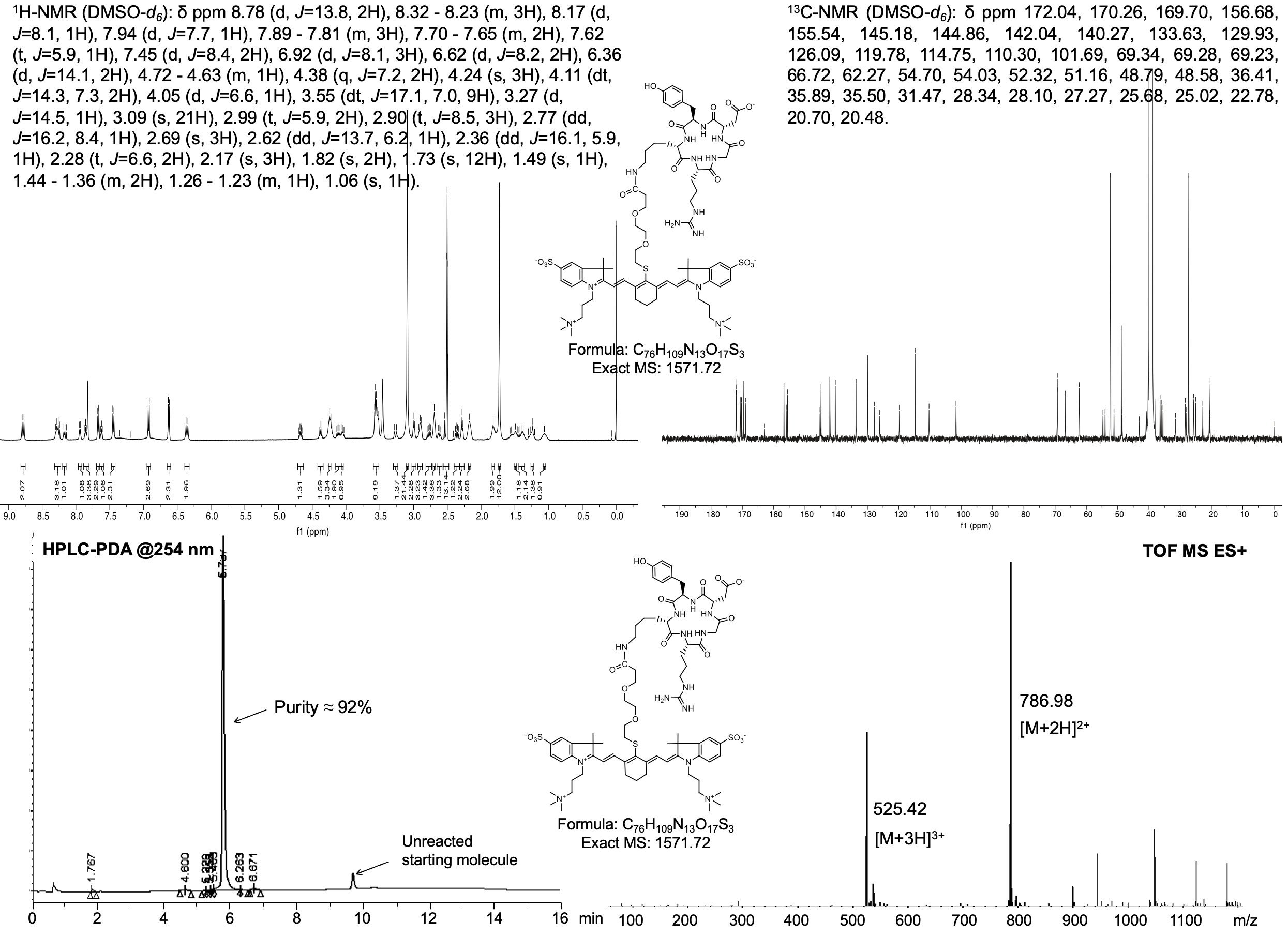


**Figure S2.** ^1^H- and ^13^C-NMR spectroscopy and HPLC-MS spectrometry of cRGD-ZW800-PEG.


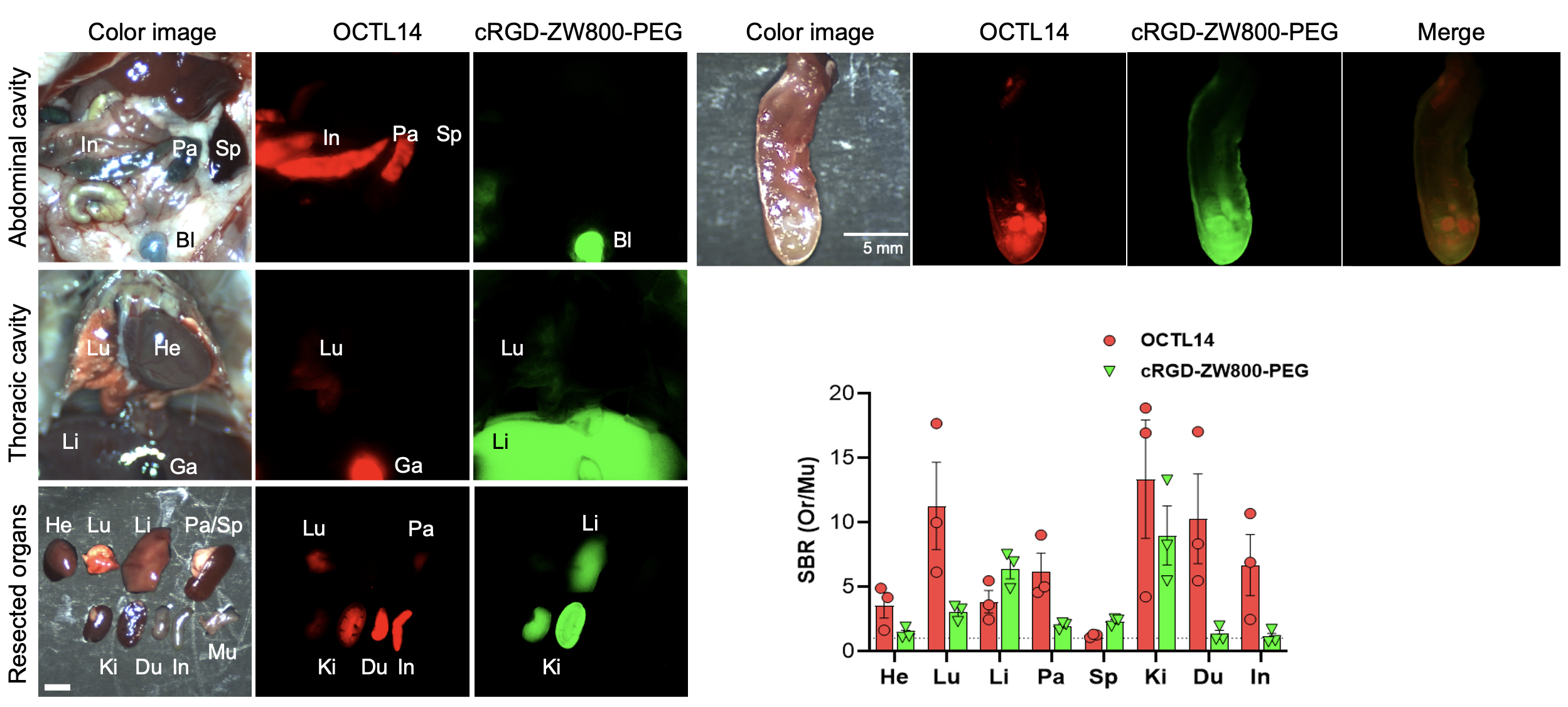


**Figure S3.** Real-time NIR fluorescence imaging of OCTL14 and cRGD-ZW800-PEG in major organs and tongue tumor. The images were acquired 4 h post-intravenous injection of OCTL14 and cRGD-ZW800-PEG into the mouse with tongue tumors. The signal-to-background ratio (SBR) of major organs was calculated using ImageJ and Prism 8 software. NIR, near-infrared; He, heart; Lu, lung; Li, liver; Pa, pancreas; Sp, spleen; Ki, kidney; Du, duodenum; In, intestine; Mu, muscle; Tu, tumor. SBR = fluorescence intensity of a region of interest / surrounding nontumor region (muscle tissue). *n* = 3. Scale bars, 5 mm.
